# An Alu Element–Associated Hypermethylation Variant of the *POMC* Gene Is Associated with Childhood Obesity

**DOI:** 10.1371/journal.pgen.1002543

**Published:** 2012-03-15

**Authors:** Peter Kuehnen, Mona Mischke, Susanna Wiegand, Christine Sers, Bernhard Horsthemke, Susanne Lau, Thomas Keil, Young-Ae Lee, Annette Grueters, Heiko Krude

**Affiliations:** 1 Institut für Experimentelle Pädiatrische Endokrinologie, Charité - Universitätsmedizin Berlin, Berlin, Germany; 2 Institut für Pathologie, Charité - Universitätsmedizin Berlin, Berlin, Germany; 3 Institut für Humangenetik, Universitätsklinikum Essen, Essen, Germany; 4 Pediatric Allergology, Experimental and Clinical Research Center, Universitätsmedizin Berlin, Berlin, Germany; 5 Institut für Sozialmedizin und Epidemiologie, Charité - Universitätsmedizin Berlin, Berlin, Germany; 6 Max Delbrück Centrum für molekulare Medizin (MDC), Berlin-Buch, Germany; University of Cambridge, United Kingdom

## Abstract

The individual risk for common diseases not only depends on genetic but also on epigenetic polymorphisms. To assess the role of epigenetic variations in the individual risk for obesity, we have determined the methylation status of two CpG islands at the *POMC* locus in obese and normal-weight children. We found a hypermethylation variant targeting individual CpGs at the intron2–exon3 boundary of the *POMC* gene by bisulphite sequencing that was significantly associated with obesity. *POMC* exon3 hypermethylation interferes with binding of the transcription enhancer P300 and reduces expression of the *POMC* transcript. Since intron2 contains Alu elements that are known to influence methylation in their genomic vicinity, the exon3 methylation variant seems to result from an Alu element–triggered default state of methylation boundary definition. Exon3 hypermethylation in the *POMC* locus represents the first identified DNA methylation variant that is associated with the individual risk for obesity.

## Author Summary

Twin studies reveal a strong genetic background of body-weight regulation. However, gene mutations in early onset obesity patients are rare. Results from large genome-wide association studies explain less than 4% of body-weight variability. Therefore, other mechanisms like epigenetic alterations may play a role in body-weight regulation. We analysed the DNA methylation of the *POMC* gene, which plays a central role in body-weight regulation within the hypothalamus. We observed a significant increase in the methylation score in obese children as compared to normal-weight individuals. This DNA methylation variant affects *POMC* gene dosage regulation. Therefore we conclude that this DNA hypermethylation variant in obese patients leads by modification of *POMC* gene expression to an increased individual risk for the development of obesity. This result illustrates how DNA methylation alterations increase the susceptibility to a common disease like obesity.

## Introduction

Inter-individual variations of epigenetic modifications like CpG methylation can alter gene function and may play a role as an individual risk for common diseases like obesity [1]. In contrast to the nucleotide sequence of DNA, the level and distribution of CpG methylation can change during development and aging as shown by the increasing differences in DNA methylation in monozygotic twins over lifetime [2], [3]. This dynamic feature of DNA methylation has focussed the interest of current research on the potential environmental impact on DNA modification as one molecular mechanism that might transmit the phenomenon of early programming [4]. However, the majority of the methylome remains remarkably stable despite aging and differences in the environment [5]–[7]. Therefore it seems likely that the methylome varies between individuals and can influence the individual risk for diseases. Since these stable patterns are established early during development and before the separation of germ layers [8], [9] they are similar in different tissues and cell types and can be identified in a representative manner by the use of easy accessible DNA from peripheral blood cells (PBC) [10].

Recent genome wide methylation studies in DNA from PBC identified the first inter-individual variations of methylation that correlate with the phenotype BMI [11]. These variants represent regions of different CpG island methylation (“variably methylated regions, VMR”) [11]. Because even a single CpG methylation difference can have a strong impact on gene transcription under some circumstances [12] a large number of small methylation differences in single CpGs might be present in the genome aside from VMRs and might account for an obese phenotype. In order to identify such obesity-associated single CpG variants we used a classical candidate gene approach. Candidate genes with potential obesity associated epigenetic variants can be deduced from patients with monogenetic obesity.

In most patients with early-onset monogenetic obesity, mutations were found that reduce the dose of signals in the anorexigenic leptin pathway within the arcuate nucleus, including the leptin-responsive gene preproopiomelanocortin (*POMC*) [13]. In addition, genome wide association studies (GWA) revealed several single nucleotide polymorphisms (SNP) among others in the *POMC* gene locus that was associated with obesity phenotypes [14]. Moreover mutations in the *POMC* gene are dependent on the gene dosage [15]. Together these genetic evidences for a dosage sensitive role of *POMC* in the pathogenesis of obesity made us to choose the *POMC* gene as a candidate to search for CpG methylation variants that might correlate with childhood obesity.

## Results

### DNA methylation of the *POMC* locus in normal weight individuals

**Figure 1:**
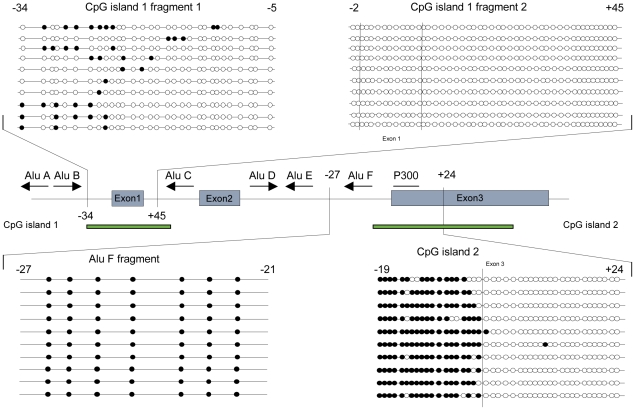
*POMC* CpG methylation pattern of CpG island 1 and 2. Analysis example of the DNA methylation pattern of human peripheral blood cells (PBC) from a normal weight individual after sequencing of 10 different clones of the PCR amplification product. Filled cycles represent methylated CpGs, open cycles non-methylated CpG positions, which are numbered according to their relative position to the start of the next exon. Alu element positions and P300 binding site are marked.

The *POMC* gene contains three exons. The functionally relevant peptides ACTH, MSH and beta-endorphin are cleaved from the C-terminal part of the protein that is translated from the exon 3 sequence [16]. Different *POMC* transcripts were described within the major population initiated via the promoter proximal to exon 1 and some smaller, less well-described variants that might be transcribed from alternative 3′ transcription start sites [17], [18]. Two CpG islands have been identified within the *POMC* gene located at the 5′-promoter-region and around the intron 2 and exon 3 boundary (Figure 1). To estimate the stability of the CpG methylation pattern within these islands, we initially compared different *POMC* expressing tissues and DNA samples taken from healthy individuals at different ages.

**Figure 2:**
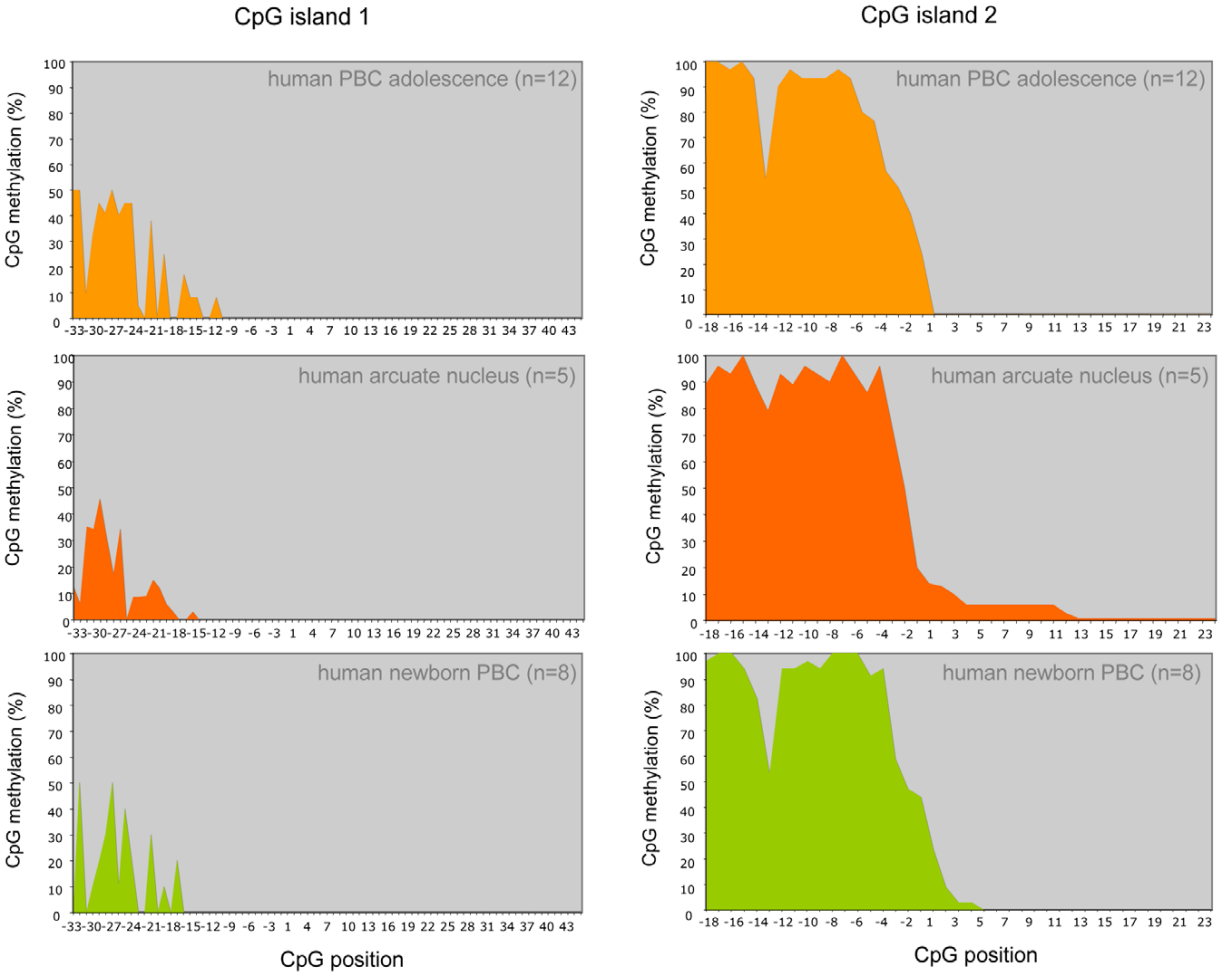
*POMC* DNA methylation pattern of CpG island 1 and 2 in normal-weight adolescents (n = 12), laser-microdissected MSH positive cells of the human arcuate nucleus (n = 5) and human newborn samples (n = 8). CpG positions are numbered according to their relative position to the next exon start.

Bisulphite sequencing of DNA samples from peripheral blood cells (PBC) of 12 normal weight adolescent individuals revealed distinct DNA methylation patterns at both CpG islands. At the 5′-promoter CpG island an overall hypomethylation was found with some reproducible methylation peaks before exon 1 which were most prominent at position −20 to −22 and −27 to −30. The second 3′ CpG island was characterized by a particularly sharp shift from hypermethylation in intron 2 to hypomethylation in exon 3 at the 3′-CpG island (Figure 1 and Figure 2).

In PBCs the *POMC* gene is expressed and its function has been analysed in different immune cells. Moreover, both, the short and long *POMC* transcript variants have been described in PBCs [19]–[21]. We subsequently investigated the *POMC* gene methylation pattern in a second cell type that expresses the gene within the context of body weight regulation. DNA was derived from MSH-positive neurons of the human hypothalamic arcuate nucleus after laser-microdissection of post-mortem hypothalamic specimen [22] (Figure 2). We found the identical pattern as in DNA from PBC. CpG island 1 was mainly hypomethylated with the same peaks of methylation at position −20 to −22 and −27 to −30 and the same sharp change in CpG island 2 of hypermethylation in intron 2 to a hypomethylation in exon 3 was present (Figure 2).

Finally we investigated the stability of *POMC* CpG-methylation during life. We compared the pattern in adolescent PBC DNA with the pattern in DNA derived from newborn (PBC-DNA from Guthrie spots). In all samples we found the identical methylation pattern in CpG island 1 and 2 irrespective of the age of the probands (Figure 2). Since we found the identical methylation pattern of *POMC* CpG islands in PBCs at different ages and in arcuate nucleus cells, both deriving from different germ layers and both expressing the *POMC* gene in different functional contexts, we concluded that the identified particular methylation pattern is set very early during development and is stable irrespective of the physiological function of the cells.

### DNA hypermethylation in obese patients

After having identified a region of stable DNA methylation in the *POMC* gene in normal healthy individuals, we set out to identify *POMC* gene methylation variants that are enriched in the obese childhood population. We compared the *POMC* gene methylation pattern in DNA derived from PBC of obese and normal weight children. The two cohorts did not differ in their age or gender distribution and the range of obesity was from +2,1 to +6,9 standard deviation scores of body mass index (SDS-BMI) [23]. Only obese children were included in whom *POMC* gene mutations had already been excluded.

**Figure 3:**
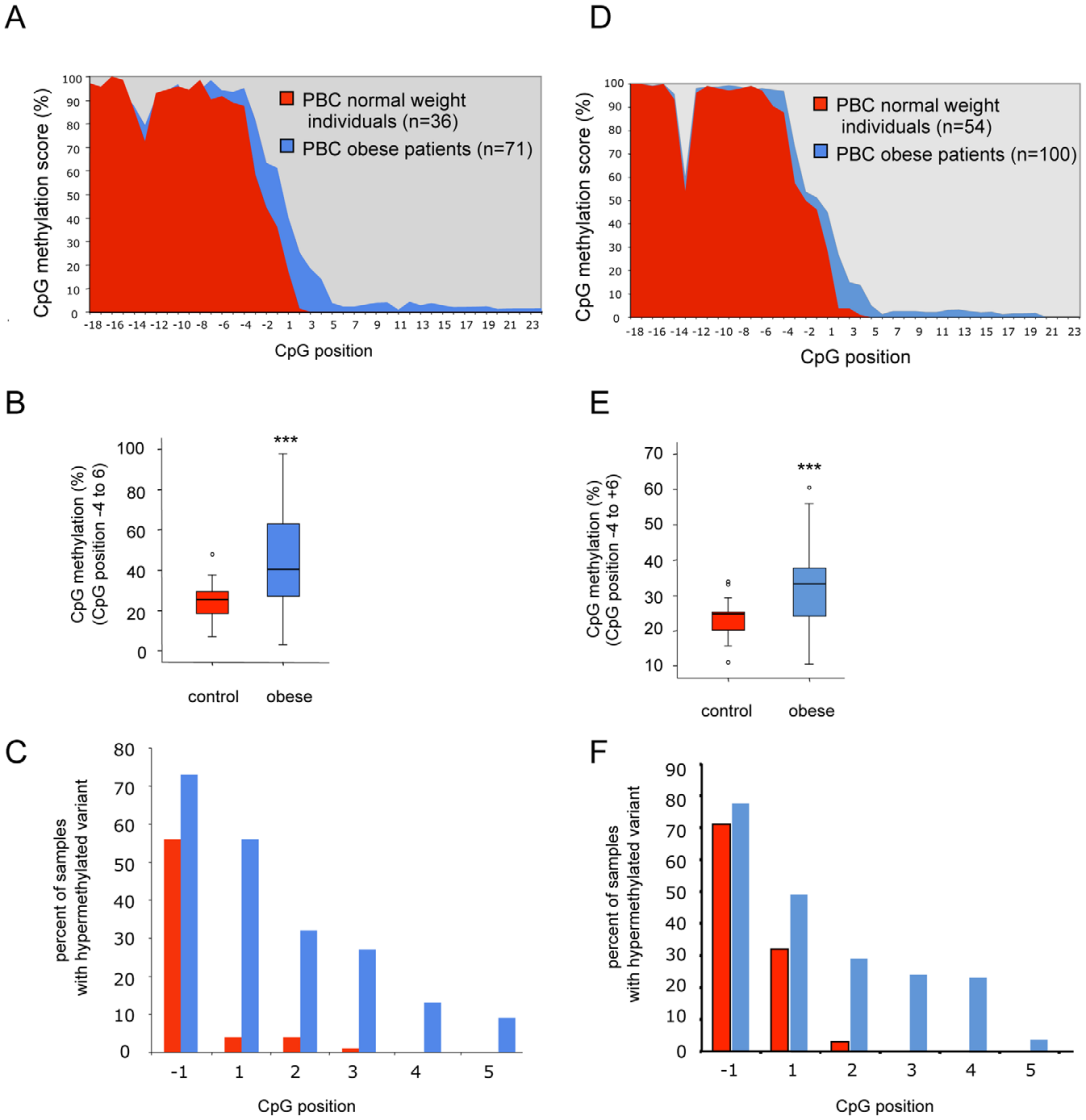
The DNA methylation was analysed with two different bisulphite based protocols in two independent cohorts. A, B, C agarose-embedded DNA was analysed by bisulphite sequencing. In D, E, F DNA was bisulphite treated in a non-agarose-bead protocol. A/D Diagram of the DNA methylation score (%) of *POMC* CpG island 2 within PBC of normal weight (red) and obese individuals (blue) (p<0,001). CpG positions are numbered according to their relative position to the next exon start. B/E Box plots analysis represents the statistic differences from the mean CpG methylation score (%) of CpG position −4 to +6 in normal weight individuals versus obese patients. C/F Percent of normal weight (red) and obese (blue) individuals, who showed DNA methylation at the annotated single CpG position (−1 to +5) in relation to the analysed number of samples.

Using DNA samples of 71 obese and 36 normal weight children we observed the identical pattern of CpG island 1 methylation in all children irrespective of their BMI (data not shown). In CpG island 2 we found a difference of methylation in obese children showing an extension of the intron 2 hypermethylation into the normally hypomethylated exon 3 (Figure 3A, Figure S1). Because we observed a certain level of variability at each CpG position in CpG island 2 in this large number of individuals we further confirmed the individual reproducibility of the methylation profiles by examining the intra-individual variability and stability of the methylation status. We analysed the DNA methylation of different PCR amplifications and different blood samples of either two normal weight or two obese individuals, in whom a hypermethylated variant had been identified (Figure S2). We found an intra-individual variability at the 3- and 5- border of differential methylated region that affects one or two single CpG positions. These intra-individual differences were smaller compared to the inter-individual differences of the two normal weight versus the two obese patients. In order to define a “hypermethylation variant” even further despite this intra-individual variability at single CpG positions, we calculated the overall methylation score of the variable region rather then analysing single CpG positions separately. We included the most variable positions of the region being the last four 3′-CpG positions of intron 2 and the first six 5′-CpG positions of exon 3 (Figure 3). The resulting overall CpG methylation score from position −4 to +6 was calculated in all children of the cohort and revealed a significant difference in obese versus normal weight children (25% versus 40%) (p<0.001) (Figure 3B). The most prominent difference of a single CpG position was found at position +1 where only 5% of normal weight children but 55% of obese children showed methylation (Figure 3C).

To confirm these results, we performed a second independent case-control study based on an alternative bisulphite sequencing protocol (direct sequencing, see Text S1) in additional 100 obese and 54 normal weight children. We found the same methylation variant of CpG island 2 with a highly significant difference in the overall methylation score of position −4 to +6 in normal weight (23%) versus obese (32%) children (p<0.001) (Figure 3D–3F).

Based on the intra-individual methylation variability and the observed differences of the overall methylation score at CpG island 2 in the two different cohorts, that might be related to and further enhanced by the different bisulphite sequencing methods, a “hypermethylation variant” versus a “hypomethylation variant” can only be defined by statistical means. We therefore calculated the percentiles of methylation score in the −4 to +6 region of CpG island 2 in the two normal weight children cohorts. We found a level of 56% methylation score as the 95^th^ percentile of overall methylation in cohort 1 and of 36% in cohort 2. Using the 95^th^ percentile of methylation score in the normal weight controls as a cut-off level for a “hypermethylation variant”, 30% of the obese children versus 5% of normal weight children within the first cohort were found to have the “hypermethylation variant”.

In order to exclude additional genetic differences that might cause the observed methylation variants, the *POMC* intron 2 - exon 3 regions were sequenced and genotyped for all known SNPs in the *POMC* locus in all individuals. No further genetic mutations could be identified in the intron 2-exon 3 region and no significant correlation between the individual SNPs and DNA methylation pattern was detected (Figure S3).

### The *POMC* hypermethylation variant is established early in life

**Figure 4:**
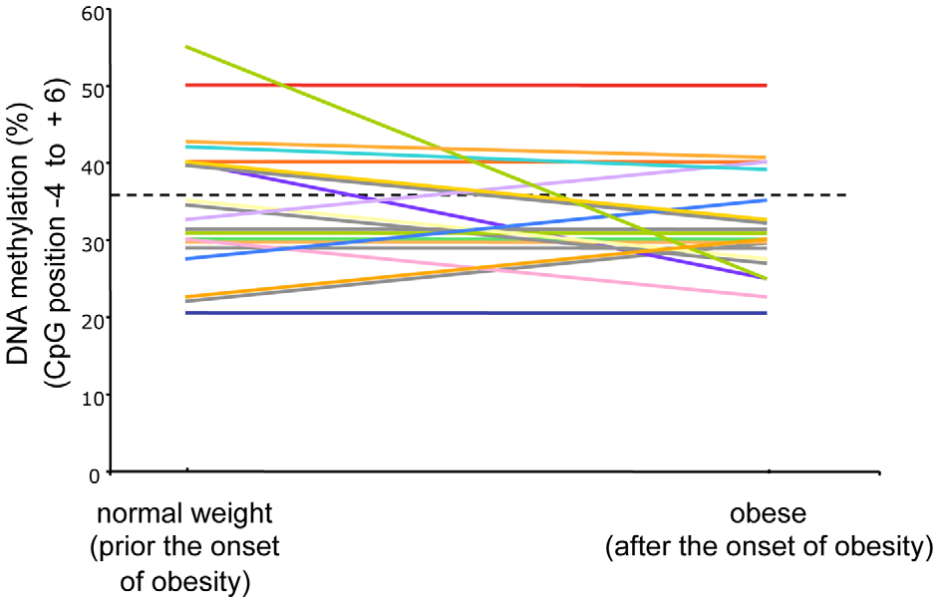
Longitudinal analysis of *POMC* DNA methylation score (%) (CpG position −4 to +6) before and after the development of obesity in altogether 21 participants of the MAS birth cohort. Each line represents one individual course of DNA methylation score prior to and after weight gain.

Several studies have indicated that DNA methylation might change during life due to environmental influences. Even though we identified identical *POMC* methylation patterns in DNA from normal weight newborns and adolescents - which is a strong argument for the stability of early established patterns - we cannot rule out that the methylation variant observed in obese children may be a consequence of obesity. Therefore, we analysed the *POMC* DNA methylation pattern in a longitudinal birth cohort (Multicenter Allergy Study, MAS) [24], which was initiated in 1990 and followed up until 2010. Participants of this study were followed from birth up to 20 years of age and DNA samples were available for the age of 5, 13 and 20 years. We focussed on those individuals, who were of normal weight at 5 and 13 years and became obese at the age of 13 or 20 years. In 21 of these cases DNA material was still available from these time points. In 8 of these individuals we identified the same *POMC* hypermethylated variant of exon 3 that we had already found in the two obese cohorts studied before. Based on the previous results we defined a “hypermethylated variant” as having a methylation score above the 95^th^ percentile of the DNA methylation of the normal weight control groups, which in our case was 36%. The cut-off value was chosen because the same bisulphite sequencing protocol was used. In all 8 individuals hypermethylation was already present prior to the development of obesity (Figure 4). In one case the DNA methylation score decreased up to the age of 20 years – however, this DNA methylation score was still in the upper range. Overall the degree of methylation (position −4 to +6) did not change during the 16 years period of obesity manifestation. These data of the MAS cohort participants strongly suggest that the hypermethylation variant of the *POMC* gene represents an early-established DNA methylation “signature” that occurs early in life in a subset of patients and might increase the risk for obesity rather than representing a consequence of obesity.

### Novel promoter activity of the *POMC* intron 2–exon 3 region in vitro

A strong association of the exon 3 hypermethylation variant with the obese phenotype suggests an influence of this variant on *POMC* gene expression or function. In principle, methylation of CpGs interferes with the accessibility of the DNA for proteins that modulate chromatin and gene transcription. In studies from the early 1990th the 3′region of the *POMC* gene including the exon 3 hypermethylation sequence was discussed to probably represent an alternative promoter for short *POMC* transcripts in different tissues [17]. Moreover, in this region a sequence fitting the guidelines of Kozak has been identified that would allow translation of a shorter protein including the functional relevant MSH peptides [17], [18], [25]. Whether short *POMC* transcripts are translated into functional peptides is still matter of a controversial debate and the results of different studies have been contradictory [21], [26]–[30]. However, in peripheral blood cells both the long and the short transcript variant have been identified [19], [20]. Despite these data the promoter activity of this intron 2-exon 3 region has not been investigated.

Therefore we analysed the promoter activity of this 3′ *POMC* region by using a *POMC* intron 2-exon3 construct in a CpG free luciferase vector [31] (kindly provided by M. Rehli, Regensburg, Germany). The functional assay was performed with a GT 1-7 hypothalamic mouse cell line. We observed an increased luciferase activity of the intron 2-exon 3 containing vector compared to the negative control, suggesting a potential promoter activity of this *POMC* region (Figure S4). Most importantly, following treatment of the 53 CpG sites containing construct with the CpG methyltransferase SssI the transcriptional activity of the intron 2-exon 3 construct was diminished. Together these data suggest a so far unrecognized transcriptional activity of the intron 2-exon 3 region within the *POMC* gene that can be inhibited by hypermethylation.

### Exon 3 hypermethylation, P300 complex binding, and *POMC* gene expression

**Figure 5:**
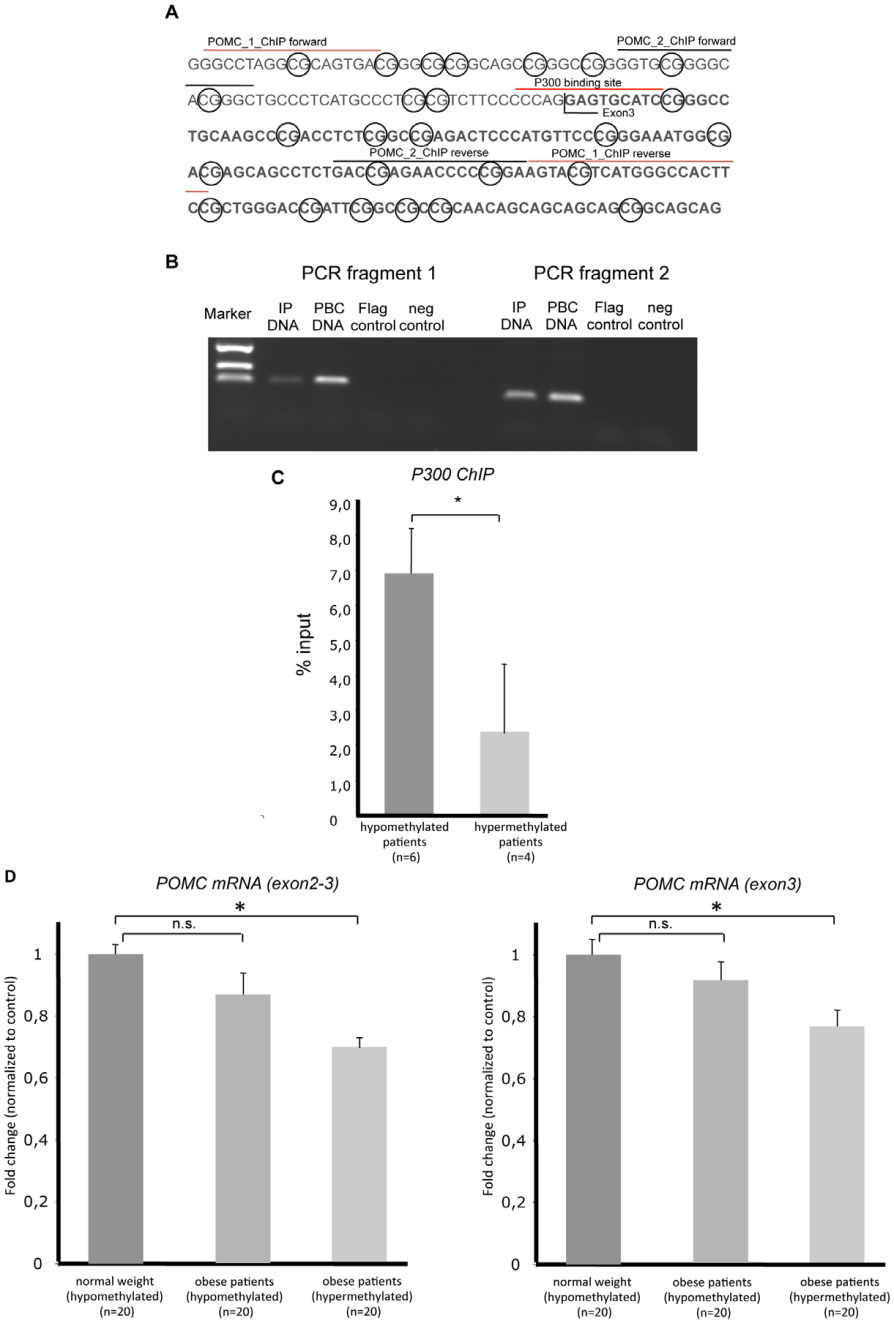
*POMC* intron2 exon3 genomic sequence, P300 ChIP assay, real-time PCR analysis, and real-time *POMC* PCR. (A) *POMC* intron2 exon3 genomic sequence with annotated ChIP primer localization and P300 binding site. (B) P300 ChIP assay was performed at three independent occasions within human peripheral blood cells. *POMC* fragment was amplified with two different primer pairs (fragment 1 and 2). Confirmation of a published P300 binding site within the insulin gene promoter in β-TC3 cells was used as a positive control [34] (Figure S5). (C) Real time PCR analysis (PCR fragment 2) of the P300 ChIP results of 4 obese patients with a hypermethylation variant and 6 individuals with hypomethylated intron 2-exon 3 intersection at *POMC* CpG island 2. (D) Real-time *POMC* PCR (*POMC* exon 2–3 and *POMC* exon3) of cDNA extracted from PBC of hypomethylated normal weight individuals (n = 20), hypermethylated obese patients (n = 20), and obese individuals without the hypermethylated variant (n = 20) reveal reduced POMC gene expression in the hypermethylated samples.

In order to further characterize a potential role for exon 3 hypermethylation in controlling *POMC* gene function we tried to identify proteins that might bind to the 3′ promoter and that could be inhibited by methylation at positions +1 to +5 of exon 3. By *in silico* analysis (http://www.cbil.upenn.edu/cgi-bin/tess/tess) we found that the intron 2 – exon 3 junction contains a putative binding site for the histone acetyltransferase P300 complex in mice and humans, which is involved in chromatin acetylation and gene activation [32], [33] (Figure 5A). In order to confirm these in silico data we performed a chromatin-immune-precipitation (ChIP) assay. A specific binding of P300 to the *POMC* exon 3 in DNA from human PBC DNA could be demonstrated (Figure 5B). We used a published P300 binding site within the insulin promoter as a positive control experiment (Figure S5) [34]. The P300 binding site in exon 3 is localized directly 3′ adjacent to the sequence with the most intense hypermethylation at CpG-position +1 in obese children (Figure 5A). Therefore we hypothesized, that hypermethylation in obese children at position +1 interferes with P300 binding and subsequently reduces *POMC* expression. We tested this hypothesis by analysing the P300 binding capacity with ChIP in PBC of 4 obese patients with the *POMC* hypermethylation variant (mean DNA methylation score 45%±4 at CpG position −4 to 6) compared to 6 normal weight individuals (mean DNA methylation score 25%±5 at CpG position −4 to 6). By real-time PCR analysis of the ChIP products, we observed a significantly reduced P300 binding (Figure 5C), indicating that hypermethylation might affect binding of the P300 complex. Because P300 is a member of an acetyltransferase complex its reduced binding in hypermethylation will influence more likely the regulation of complex chromatin function rather then local promoter activity of the intron-2 region. To asses the impact of the hypermethylation variant on *POMC* gene expression we performed real time RT-PCR of *POMC* products from PBC in 20 hypermethylated obese patients (mean DNA methylation score 48%±6 at CpG position −4 to 6) compared to 20 normal weight control individuals (mean DNA methylation score 25%±5 at CpG position −4 to 6) and 20 obese patients without the *POMC* hypermethylated variant (mean DNA methylation score 30%±5 at CpG position −4 to 6). In PBC with the hypermethylation variant we observed a significantly reduced PCR product of a fragment that covers exon3 alone as well as a fragment that covers exon2 and exon3 (Figure 5D). Because the longer fragment, that covers exon2 and exon3 represents a transcript that is transcribed from the 5′ promoter of the POMC gene, we conclude that the hypermethylation variant interfere at least in part with the transcription of the functionally relevant and well known long transcript containing the signal peptide and the coding region for the melanocortin peptides. However, whether an additional influence of the hypermethylation variant on a shorter 3′ transcript exists, cannot be excluded.

### Potential molecular origin of the exon 3 *POMC* hypermethylation variant

The results of our functional assays suggest that P300 binding to the 5′ sequence of exon 3 is critical for *POMC* gene expression and that methylation of exon 3 CpGs seems to interfere with P300 binding. Consequently we hypothesized that for *POMC* gene expression exon 3 CpGs need to remain hypomethylated in different species that contain a P300 binding site around CpG position +1. To verify this assumption we investigated the methylation status of the second CpG island as well as the nucleotide sequence of the intron 2-exon 3 region in mice.

**Figure 6:**
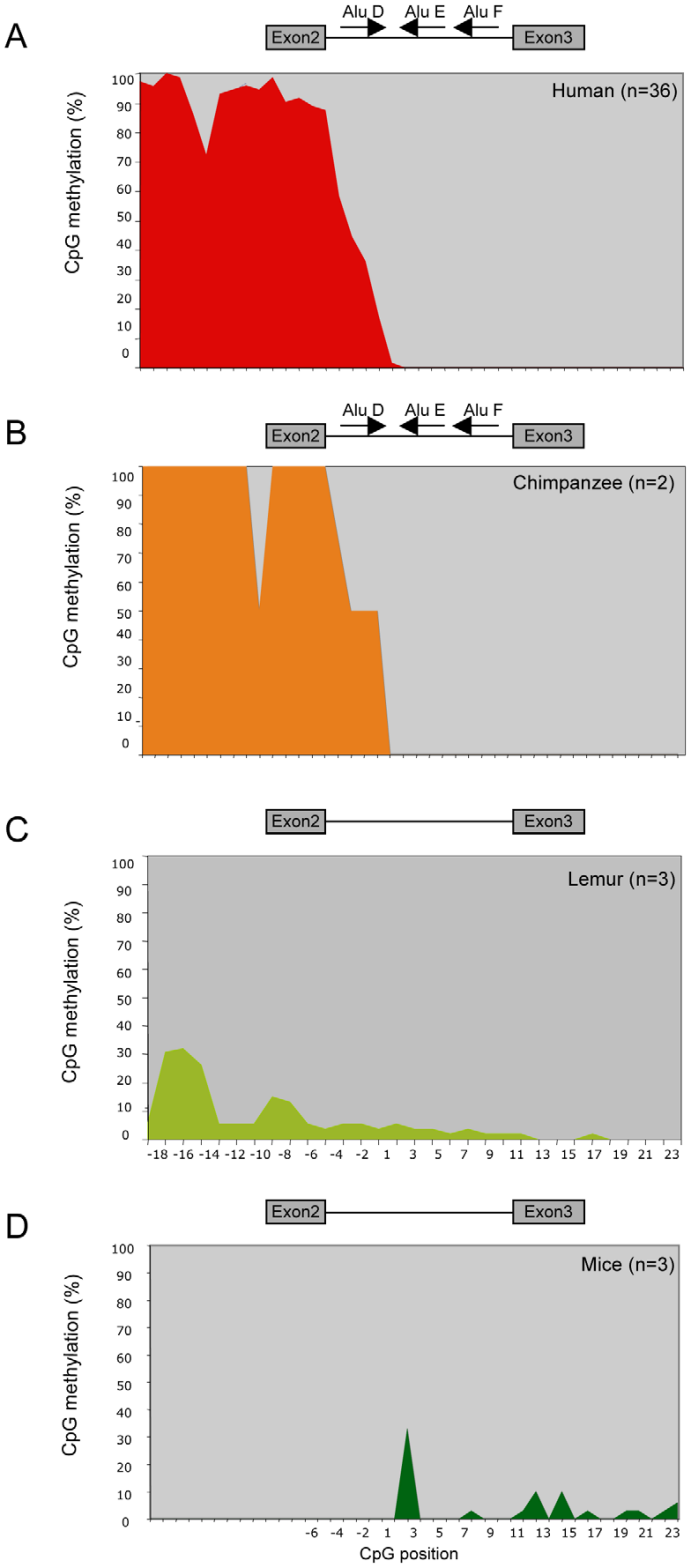
Species-specific *POMC* methylation pattern *POMC* DNA methylation pattern after bisulphite genomic sequencing of CpG island 2. In (A) normal-weight human individuals (n = 36), (B) chimpanzee PBC (n = 2), (C) lemur PBC (n = 3), and (D) mice PBC (n = 3). CpG positions are numbered according to their relative position to the exon 3 start.

Comparison of the human and mice *POMC* locus revealed the predicted phylogenetically conserved hypomethylation of exon 3 in mice. Surprisingly, in contrast to the human methylation pattern, the murine intron 2-sequence is not methylated at all (Figure 6A, 6D). Further comparison of the murine and human intron 2 sequences revealed a difference in the locus structure in that the human intron 2 contains three Alu elements (Figure 1), which are not present in mice [35]. The presence of Alu elements within intron 2 in our cohorts was confirmed by sequencing analysis of all samples (data not shown). Because Alu elements are known to alter the methylation pattern in their genomic vicinity [36], [37] we further hypothesized that the *POMC* intron 2 hypermethylation in humans is caused by the presence of the Alu elements within intron 2. We tested this hypothesis by bisulphite sequencing of the *POMC* intron 2-exon 3 region from different primates, since Alu elements are only present in primate genomes, however to a different extent [38]. We found, that primates - like the chimpanzees - with the same Alu elements as the human sequence share the same human DNA methylation pattern of intron 2 hypermethylation and exon 3 hypomethylation (Figure 6B). In contrast, in DNA from more distant primates - like lemurs - who do not contain Alu elements in intron 2, we found a complete hypomethylation of intron 2 (Figure 6C) as in the Alu element-free mouse *POMC* gene.

These data from mouse, human, and other primate *POMC* gene loci show a close association between the presence of Alu elements in intron 2 with the state of hypermethylation of intron 2 CpGs suggesting that the Alu elements in intron 2 may trigger methylation. Because hypomethylation of exon 3 seems to be critical for *POMC* gene expression one can speculate, that in species with Alu elements the hypomethylation state of exon 3 needs to be defended against an Alu element triggered intron 2 methylation force. In this scenario Alu element triggered intron 2 methylation needs to be restricted with a sharp border at the first 5′ CpGs of exon 3. The hypermethylation variant of these first 5′-CpGs of exon 3 would therefore represent a default state of methylation restriction with an extension of intron 2 methylation into exon3.

## Discussion

During the last few years a large number of highly powered GWA studies were conducted to identify genetic variants that correlate with an increased genetic risk for obesity [14]. Despite the successful identification of several SNPs that influence the individual body weight, their overall impact on the weight phenotype variation was estimated to be less than 4% [39], [40]. Therefore, other inter-individual variants of the chromatin like epigenetic polymorphisms need to be considered as a further determinant for the individual body weight phenotype.

*POMC* methylation has been analysed in different studies before. Hence, a methylation dependent *POMC* promoter activity has been described *in-vitro* [41]. Moreover altered *POMC* promoter methylation has been observed in different tumor cell-lines, which was accompanied by changes in *POMC* gene expression [41], [42]. Finally, reduced POMC methylation has been identified in fetal hypothalamic samples from offspring after maternal undernutrition in sheep, which was attended by increased *POMC* gene expression [43], [44]. The latter study provides the most explicit evidence for an interrelation between metabolism and epigenetic regulation of the *POMC* gene in mice, which was further supported recently in another animal study [45].

We now describe the first epigenetic variant in the *POMC* gene in humans being highly associated with severe obesity in children. The hypermethylation variant affects only a few distinct single CpG-sites at the border of a hypermethylation stretch in intron 2. Upon increased methylation in obese children, methylation extended this CpG-hypermethylated region into the non-methylated exon 3.

Hypomethylation of exon 3 is highly conserved in different species suggesting a functional relevance of an open chromatin state of this *POMC* region. Accordingly, we could demonstrate a reduced binding of the transcription enhancer P300 to its binding site in direct proximity to the methylation variant and we could show a reduced level of POMC transcripts in PBC with the hypermethylation variant. Moreover the hypermethylation variant leads to a reduced mRNA expression of the long *POMC* transcript containing exon2 and exon3, which suggests that the 5′ promoter of the POMC gene can be indirectly influenced by the methylation state of the intron2-exon3 3′ region. Together these findings argue that the intron2-exon3 region might function as an enhancer for the 5′ *POMC* promoter transcription.

Genome-wide studies of DNA methylation patterns showed a reduced methylation density at P300 binding sites. The latter are predominantly located at gene activating elements and display an increased monomethylation of H3K4, which indicates an open chromatin formation [33], [46]. However, a transcriptional regulatory activity of the intron 2-exon 3 region of the *POMC* gene was not described before, despite the fact that several studies have identified smaller *POMC* transcripts. These are not transcribed from the 5′ *POMC* promoter, but are rather likely to originate from the intron 2 region. We could now show a transcription activity of the intron 2-exon 3 fragment of the human *POMC* gene in a luciferase reporter construct. The activity could be blocked by pre-treatment with the CpG methyltransferase SssI. Together these data strongly suggest that the *POMC* gene region containing the hypermethylation variant is relevant for *POMC* gene transcription and that methylation can interfere with this transcriptional activity and function of the *POMC* gene. Because we could further show a reduced expression of the long transcript containing exon2 and exon3 it seems reasonable that the hypermethylation variant reduces an enhancer activity of the intron2-exon3 region that act on the 5′ promoter. Therefore the association of the hypermethylation variant with obesity results from a reduced expression of the functional relevant long *POMC* transcript containing the signal peptide and the weight regulating melanocortin peptides by interfering with a 3′ enhancer of the *POMC* gene. However, it cannot be excluded that in addition a shorter *POMC* transcript that originates from the intron2-exon3 region is affected by the hypermethylation variant.

The CpG methylation variant is associated with body weight, but was detected in DNA from peripheral blood leucocytes, which do not participate in weight regulation. Therefore, it is tempting to speculate that the variant in blood cells reflects a non tissue-specific general methylation pattern that is also present within *POMC* expressing cells in which it plays a role in the weight regulatory pathway. We have shown that the overall methylation pattern of CpG island 2 is very stable in different cell types, including *POMC* expressing cells of the arcuate nucleus as well as during the individual lifetime and in different primate species. Hence, the individual pattern of CpG island 2 methylation seems to be established early during development and remains stable in different cell types irrespective of age and environment. We postulate that this stability within different cells and lifetime periods also exists for the obesity-associated hypermethylation variant of CpG island 2, because obese individuals with the variant have been shown to harbour the hypermethylation pattern of CpG island 2 already before the onset of obesity at a young age. However, due to obvious ethical reasons we could not finally prove the presence of the variant in the critical hypothalamic arcuate nucleus neurons of obese patients.

While hypomethylation of exon 3 is conserved in all species tested, including mice, we found the hypermethylation of intron 2 only in the genomes of primates who contain Alu-elements. Alu elements derive from retrotransposons and are known to trigger methylation in their genomic vicinity presumably to reduce transcriptional activity of the retrotransposomal DNA [47]. It is tempting to speculate that the methylation of Alu elements and other retrotransposons during early development may stochastically spread into adjacent regions. This is similar to position-effect variegation in *Drosophila*, although over much shorter distances, and these patterns are then stably transmitted to daughter cells during further development [48]. In this respect, the hypermethylation variant in *POMC* exon 3 would result from an Alu-triggered methylation force that is usually restricted to intron 2, but may extend to the first CpG of exon 3 in terms of a border demarcation weakness. Due to the transcriptional activity of this exon 3 sequence the Alu-triggered hypermethylation may affect *POMC* expression and influence the risk for obesity. Such epigenetically controlled transcriptional interference by transposons has been described before in several inherited diseases such as X-linked dystonia-parkinsonism [49] and in several organisms [50], including the agouti viable yellow mice [51]. In these examples the transposon changed its position and represents a genetic first hit that is followed by the epigenetic secondary change with pathological consequences. However, the identified *POMC* hypermethylation variant was not associated with a genomic rearrangement of the Alu elements or with any other genetic difference. From that point of view we presume that this *POMC* methylation variant is a stochastically occurring mistake in the termination of Alu element-triggered methylation leading to a potentially pathologic individual “epigenetic signature”. The molecular mechanism that regulates the exact border restriction of CpG-methylation is unknown. Therefore we cannot exclude, that another genetic variant exists in trans, outside the *POMC* locus, that interferes with an appropriate methylation border restriction and thus may influence the border definition of intron 2-methylation in terms of a primary genetic defect.

An epigenetic contribution to the pathogenesis of obesity has been widely discussed and predicted. In our study we demonstrate now for the first time an epigenetic variant of CpG methylation in the candidate gene *POMC* that seems to be functionally relevant for gene expression and that is enriched in obese children compared to normal weight individuals.

## Materials and Methods

### Ethics statement

All clinical investigations and genetic analysis were performed according the guidelines of the Declaration of Helsinki and with the agreement of all family members and the ethic committee of the Charité Virchow-Klinikum.

### Patients, controls, post-mortem brain samples, cell lines, mice, and primate DNA samples

The DNA samples of patients were collected at the obesity outpatient clinic of the Institute of Experimental Paediatric Endocrinology at the Charité Children's Hospital in Berlin.

DNA, extracted from human peripheral blood cells (Qiagen), was analysed from normal weight individuals (55 female and 35 male, average age 17,9 years, average BMI = 18,59 ^kg^/_m_^2^), and in obese children in whom *POMC* gene mutation was excluded (91 female and 80 male, average age 11 years, average BMI = 32,3 ^kg^/_m_^2^). Cohort 1: 36 normal weight individuals (female∶male = 25∶11, average age 18,4 years, average BMI 19 ^kg^/_m_^2^); 71 obese individuals (female∶male = 35∶36, average age 13,2 years, average BMI 31,2 ^kg^/_m_^2^); cohort 2: 54 normal weight individuals (female∶male = 30∶24, average age 17,4 years, average BMI 18,2 ^kg^/_m_^2^); 100 obese individuals (female∶male = 56∶44, average age 8,8 years, average BMI 33,4 ^kg^/_m_^2^). DNA extracted from PBC of 21 participants of the MAS birth cohort [24] was analysed at the age of 5 or 13 years of age (average BMI = 16,04 ^kg^/_m_^2^) before the development of obesity and at the age of 13 or 20 years (average BMI = 27,49 ^kg^/_m_^2^) after the onset of obesity longitudinally. Rolland-Cachera-BMI percentiles were used to define obesity as a BMI above the 97. percentile [23]. DNA from newborn screening cards was extracted according to standard protocols (Promega). For the analysis of the newborn blood samples parents gave special informed consent.

Brain samples were obtained from autopsies in a period of <24 h post-mortem, which were performed according to the law of Berlin (Sektionsgesetz, Gesetz- und Verordnungblatt für Berlin, 1996; 52 32, 237–239). Subjects were selected with no history of neurological diseases (2 female, 3 male, average age 73,4 years, average BMI = 29,6 ^kg^/_m_^2^).

The pancreatic cell line β-TC3 was kindly provided by the group of J. Seufert (Freiburg, Germany). β-TC3-cells and GT 1-7 cells, were maintained in DMEM-medium (Invitrogen) supplemented with 10% fetal bovine serum, penicillin (100 U/ml), streptomycin (100 µg/ml) and l-glutamin (2 mM).

Mice peripheral blood cells have been obtained from a C57BL/6 mice strain (kindly provided by J. Koehrle, Berlin, Germany). Primate blood samples have been obtained from the Deutsches Primatenzentrum (DPZ, Göttingen, Germany).

### DNA methylation analysis

DNA methylation was analysed according to standard protocols [52] with agarose embedded DNA or in the second independent cohort with direct bisulphite treated DNA. Details of both methods and cohorts are available in Text S1. The laser-microdissected cells were analysed as described [53].

### Postmortem human brain tissue, immunohistochemistry, and laser microdissection

Human brain samples were cut into 10 µm sections and stained with the beta-MSH antibody (G. Brabant, Birmingham, UK). Immunohistochemical analysis of Beta-MSH of human arcuate nucleus was carried out as previously described [22]. Further information is available in . MSH positives cells of the arcuate nucleus were extracted by laser microdissection (ZEISS).

### Luciferase reporter-gene assay

The luciferase assays were performed according standard protocols (Dual luciferase reporter assay, Promega) with a pCpGL-vector (kindly gift from M. Rehli, Regensburg, Germany) [31] in GT1-7 mouse hypothalamic cells. The POMC CpG island 2 fragment was created with a BamH1 and Nco1 restriction recognition site. *In vitro* methylation was performed with a 16 h incubation of the transfected construct (0.5 µg) with Sss1 (NEB, Promega). The transfected construct without added SAM to the enzymatic reaction serves as a negative control. Primer sequences are available in the Text S1.

### Chromatin immunoprecipitation (ChIP)

ChIP experiments were performed with DNA extracted from peripheral blood cells of patients with and without the hypermethylated *POMC* variant. Samples were preincubated with magnetic beads (Invitrogen) for 1 h (+4°C). Thereafter the samples were transferred to P300 antibody (N-15, Santa Cruz) labelled magnetic beads and incubated overnight on a tilting table. Finally the samples were incubated for 16 hours at 65°C and the final PCR was performed with two different primer pairs after ethanol washing steps. As a negative control the samples were also incubated with magnetic beads and anti-FLAG antibody (SIGMA) as well as non-antibody labelled magnetic beads. For detailed description of the ChIP protocol see Text S1.

### Quantitative real-time PCR

RNA was extracted from 2 ml EDTA blood samples using the Trizol method. These samples were collected prospectively from 20 normal weight controls and 20 obese individuals with or without the hypermethylation variant. DNAse digestion and cDNA synthesis was performed according standard protocols (NEB, Promega). Quantitative real time PCR reactions for *ß-actin* as a housekeeping gene and *POMC* were established according to standard protocols. For detailed description of the quantitative real time PCR and primer sequence see Text S1.

### Statistical analysis

To compare the degree of DNA methylation score between obese and non-obese persons the mean DNA methylation of CpG for the positions −4 to +6 was used as a test variable in a t-Test. A linear regression model with the mean DNA methylation of CpG for the positions −4 to +6 as dependent variable and age, sex and BMI standard deviation score BMI-SDS as independent variables were calculated in order to estimate the influence of BMI-SDS on mean DNA methylation of CpG positions −4 to +6.

## Supporting Information

Figure S1 A Sequence example with genomic sequence of a hypomethylated *POMC* intron 2 exon 3 boundary of a normal weight individual (B) and an obese patient with a hypermethylated *POMC* variant (C).
(PDF)

Figure S2 Analysis of the intra-individual variability of the POMC DNA methylation at the intron2-exon3 intersection. DNA methylation was analysed from two normal weight individuals and two obese individuals with the hypermethylated variant. The DNA was extracted from three different blood samples of each individual (A, B, C) and three independent PCR amplification reactions were performed with each DNA sample.
(PDF)

Figure S3 Diagram of the observed SNPs (No1-6) within the *POMC* gene in correlation to the observed DNA methylation intensity at CpG position −4 to +6 of the individual respectively.
(PDF)

Figure S4 Luciferase reporter-gene assay with a POMC CpG island 2 fragment. A schematic display of the fragment localization B Luciferase assay with empty pCpGL vector, pCpGL_CpG island 2 vector treated with Sss1 or treated with Sss1 without SAM addition as a control. The luciferase activity is annotated according the luciferase/renilla ratio of 3 independent experiments.
(PDF)

Figure S5 P300 ChIP analysis of the insulin promoter with samples of β-TC3 beta-cells. We used this published P300 binding site [34] as positive control for the established ChIP assay.
(PDF)

Text S1 A summary of the different bisulphite sequencing protocols used in this study. Moreover the primer pair sequences and the different protocols for ChIP and qPCR experiments are described in detail.
(DOC)

## References

[1] Bjornsson HT, Fallin MD, Feinberg AP (2004). An integrated epigenetic and genetic approach to common human disease.. Trends Genet.

[2] Boks MP, Derks EM, Weisenberger DJ, Strengman E, Janson E (2009). The relationship of DNA methylation with age, gender and genotype in twins and healthy controls.. PLoS ONE.

[3] Fraga MF, Ballestar E, Paz MF, Ropero S, Setien F (2005). Epigenetic differences arise during the lifetime of monozygotic twins.. Proc Natl Acad Sci U S A.

[4] Jirtle RL, Skinner MK (2007). Environmental epigenomics and disease susceptibility.. Nat Rev Genet.

[5] Bjornsson HT, Sigurdsson MI, Fallin MD, Irizarry RA, Aspelund T (2008). Intra-individual change over time in DNA methylation with familial clustering.. JAMA.

[6] Bollati V, Schwartz J, Wright R, Litonjua A, Tarantini L (2009). Decline in genomic DNA methylation through aging in a cohort of elderly subjects.. Mech Ageing Dev.

[7] Gronniger E, Weber B, Heil O, Peters N, Stab F (2010). Aging and chronic sun exposure cause distinct epigenetic changes in human skin.. PLoS Genet.

[8] Borgel J, Guibert S, Li Y, Chiba H, Schubeler D (2010). Targets and dynamics of promoter DNA methylation during early mouse development.. Nat Genet.

[9] Reik W, Dean W, Walter J (2001). Epigenetic reprogramming in mammalian development.. Science.

[10] Talens RP, Boomsma DI, Tobi EW, Kremer D, Jukema JW (2010). Variation, patterns, and temporal stability of DNA methylation: considerations for epigenetic epidemiology.. FASEB J.

[11] Feinberg AP, Irizarry RA, Fradin D, Aryee MJ, Murakami P (2010). Personalized epigenomic signatures that are stable over time and covary with body mass index.. Sci Transl Med.

[12] Martinowich K, Hattori D, Wu H, Fouse S, He F (2003). DNA methylation-related chromatin remodeling in activity-dependent BDNF gene regulation.. Science.

[13] Krude H, Biebermann H, Luck W, Horn R, Brabant G (1998). Severe early-onset obesity, adrenal insufficiency and red hair pigmentation caused by POMC mutations in humans.. Nat Genet.

[14] Speliotes EK, Willer CJ, Berndt SI, Monda KL, Thorleifsson G (2010). Association analyses of 249,796 individuals reveal 18 new loci associated with body mass index.. Nat Genet.

[15] Farooqi IS, Drop S, Clements A, Keogh JM, Biernacka J (2006). Heterozygosity for a POMC-null mutation and increased obesity risk in humans.. Diabetes.

[16] Takahashi H, Hakamata Y, Watanabe Y, Kikuno R, Miyata T (1983). Complete nucleotide sequence of the human corticotropin-beta-lipotropin precursor gene.. Nucleic Acids Res.

[17] Lacaze-Masmonteil T, de Keyzer Y, Luton JP, Kahn A, Bertagna X (1987). Characterization of proopiomelanocortin transcripts in human nonpituitary tissues.. Proc Natl Acad Sci U S A.

[18] Gardiner-Garden M, Frommer M (1994). Transcripts and CpG islands associated with the pro-opiomelanocortin gene and other neurally expressed genes.. J Mol Endocrinol.

[19] Ehrlich S, Weiss D, Burghardt R, Infante-Duarte C, Brockhaus S (2010). Promoter specific DNA methylation and gene expression of POMC in acutely underweight and recovered patients with anorexia nervosa.. J Psychiatr Res.

[20] Andersen GN, Hagglund M, Nagaeva O, Frangsmyr L, Petrovska R (2005). Quantitative measurement of the levels of melanocortin receptor subtype 1, 2, 3 and 5 and pro-opio-melanocortin peptide gene expression in subsets of human peripheral blood leucocytes.. Scand J Immunol.

[21] Buzzetti R, McLoughlin L, Lavender PM, Clark AJ, Rees LH (1989). Expression of pro-opiomelanocortin gene and quantification of adrenocorticotropic hormone-like immunoreactivity in human normal peripheral mononuclear cells and lymphoid and myeloid malignancies.. J Clin Invest.

[22] Biebermann H, Castaneda TR, van Landeghem F, von Deimling A, Escher F (2006). A role for beta-melanocyte-stimulating hormone in human body-weight regulation.. Cell Metab.

[23] Rolland-Cachera MF, Cole TJ, Sempe M, Tichet J, Rossignol C (1991). Body Mass Index variations: centiles from birth to 87 years.. Eur J Clin Nutr.

[24] Bergmann RL, Bergmann KE, Lau-Schadensdorf S, Luck W, Dannemann A (1994). Atopic diseases in infancy. The German multicenter atopy study (MAS-90).. Pediatr Allergy Immunol.

[25] Jeannotte L, Burbach JP, Drouin J (1987). Unusual proopiomelanocortin ribonucleic acids in extrapituitary tissues: intronless transcripts in testes and long poly(A) tails in hypothalamus.. Mol Endocrinol.

[26] Slominski A, Wortsman J, Luger T, Paus R, Solomon S (2000). Corticotropin releasing hormone and proopiomelanocortin involvement in the cutaneous response to stress.. Physiol Rev.

[27] Clark AJ, Lavender PM, Coates P, Johnson MR, Rees LH (1990). In vitro and in vivo analysis of the processing and fate of the peptide products of the short proopiomelanocortin mRNA.. Mol Endocrinol.

[28] Millington WR, Rosenthal DW, Unal CB, Nyquist-Battie C (1999). Localization of pro-opiomelanocortin mRNA transcripts and peptide immunoreactivity in rat heart.. Cardiovasc Res.

[29] Taherzadeh S, Sharma S, Chhajlani V, Gantz I, Rajora N (1999). alpha-MSH and its receptors in regulation of tumor necrosis factor-alpha production by human monocyte/macrophages.. Am J Physiol.

[30] Johansson O, Virtanen M, Hilliges M, Hansson LO (1991). Gamma-melanocyte-stimulating hormone-like immunoreactivity in blood cells of human eosinophilic patients.. Acta Haematol.

[31] Klug M, Rehli M (2006). Functional analysis of promoter CpG methylation using a CpG-free luciferase reporter vector.. Epigenetics.

[32] Liu X, Wang L, Zhao K, Thompson PR, Hwang Y (2008). The structural basis of protein acetylation by the p300/CBP transcriptional coactivator.. Nature.

[33] Heintzman ND, Stuart RK, Hon G, Fu Y, Ching CW (2007). Distinct and predictive chromatin signatures of transcriptional promoters and enhancers in the human genome.. Nat Genet.

[34] Chakrabarti SK, Francis J, Ziesmann SM, Garmey JC, Mirmira RG (2003). Covalent histone modifications underlie the developmental regulation of insulin gene transcription in pancreatic beta cells.. J Biol Chem.

[35] Tsukada T, Watanabe Y, Nakai Y, Imura H, Nakanishi S (1982). Repetitive DNA sequences in the human corticotropin-beta-lipotrophin precursor gene region: Alu family members.. Nucleic Acids Res.

[36] Liu WM, Schmid CW (1993). Proposed roles for DNA methylation in Alu transcriptional repression and mutational inactivation.. Nucleic Acids Res.

[37] Kochanek S, Renz D, Doerfler W (1993). DNA methylation in the Alu sequences of diploid and haploid primary human cells.. EMBO J.

[38] Xing J, Witherspoon DJ, Ray DA, Batzer MA, Jorde LB (2007). Mobile DNA elements in primate and human evolution.. Am J Phys Anthropol.

[39] Loos RJ, Lindgren CM, Li S, Wheeler E, Zhao JH (2008). Common variants near MC4R are associated with fat mass, weight and risk of obesity.. Nat Genet.

[40] Li S, Zhao JH, Luan J, Luben RN, Rodwell SA (2010). Cumulative effects and predictive value of common obesity-susceptibility variants identified by genome-wide association studies.. Am J Clin Nutr.

[41] Newell-Price J, King P, Clark AJ (2001). The CpG island promoter of the human proopiomelanocortin gene is methylated in nonexpressing normal tissue and tumors and represses expression.. Mol Endocrinol.

[42] Ye L, Li X, Kong X, Wang W, Bi Y (2005). Hypomethylation in the promoter region of POMC gene correlates with ectopic overexpression in thymic carcinoids.. J Endocrinol.

[43] Stevens A, Begum G, Cook A, Connor K, Rumball C (2010). Epigenetic changes in the hypothalamic proopiomelanocortin and glucocorticoid receptor genes in the ovine fetus after periconceptional undernutrition.. Endocrinology.

[44] Stevens A, Begum G, White A (2011). Epigenetic changes in the hypothalamic pro-opiomelanocortin gene: a mechanism linking maternal undernutrition to obesity in the offspring?. Eur J Pharmacol.

[45] Plagemann A, Harder T, Brunn M, Harder A, Roepke K (2009). Hypothalamic proopiomelanocortin promoter methylation becomes altered by early overfeeding: an epigenetic model of obesity and the metabolic syndrome.. J Physiol.

[46] Lister R, Pelizzola M, Dowen RH, Hawkins RD, Hon G (2009). Human DNA methylomes at base resolution show widespread epigenomic differences.. Nature.

[47] Li Y, Zhu J, Tian G, Li N, Li Q (2010). The DNA methylome of human peripheral blood mononuclear cells.. PLoS Biol.

[48] Muller HJ, Altenburg E (1930). The Frequency of Translocations Produced by X-Rays in Drosophila.. Genetics.

[49] Makino S, Kaji R, Ando S, Tomizawa M, Yasuno K (2007). Reduced neuron-specific expression of the TAF1 gene is associated with X-linked dystonia-parkinsonism.. Am J Hum Genet.

[50] Martin A, Troadec C, Boualem A, Rajab M, Fernandez R (2009). A transposon-induced epigenetic change leads to sex determination in melon.. Nature.

[51] Morgan HD, Sutherland HG, Martin DI, Whitelaw E (1999). Epigenetic inheritance at the agouti locus in the mouse.. Nat Genet.

[52] Hajkova P, el-Maarri O, Engemann S, Oswald J, Olek A (2002). DNA-methylation analysis by the bisulfite-assisted genomic sequencing method.. Methods Mol Biol.

[53] Kerjean A, Vieillefond A, Thiounn N, Sibony M, Jeanpierre M (2001). Bisulfite genomic sequencing of microdissected cells.. Nucleic Acids Res.

