# Supplementary material for: An Alu Element–Associated Hypermethylation Variant of the POMC Gene Is Associated with Childhood Obesity
Source: PLoS Genet. 2012 Mar 15;8(3):e1002543. doi: 10.1371/journal.pgen.1002543 (PMC3305357; doi:10.1371/journal.pgen.1002543)

A Genomic sequence

CCAGGAGTGCATCCGGGCCTGCAAGCCCGACCTCTCGGCCGA

Exon 3 start

B Normal weight individual

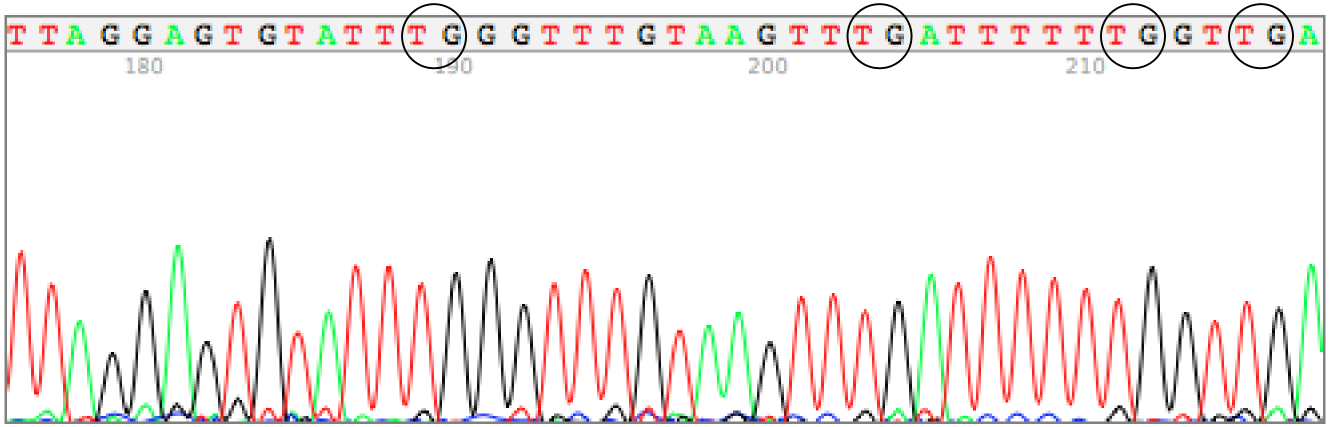

C Obese patient

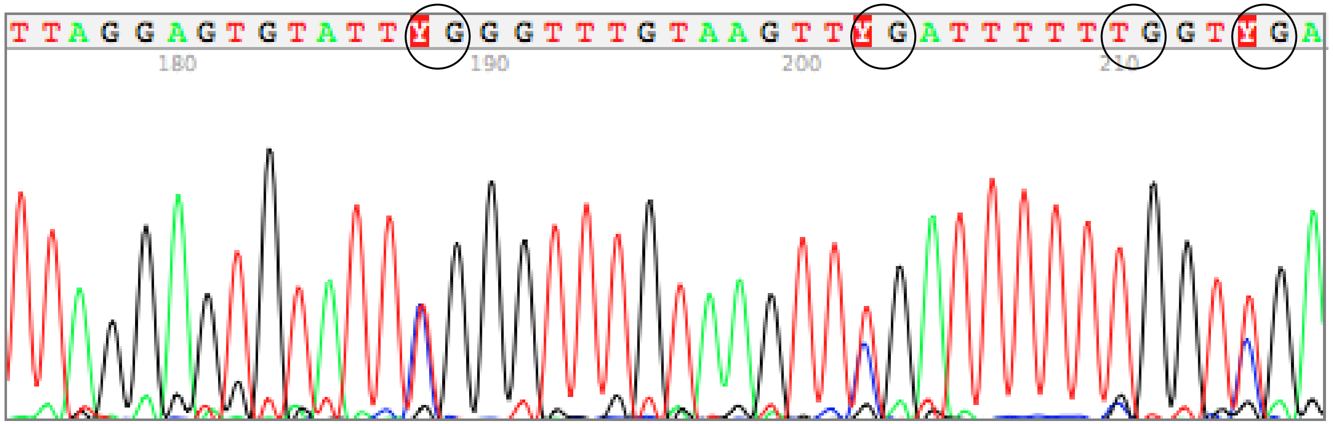

Supplement: Figure S1 — A Sequence example with genomic sequence of a hypomethylated POMC intron 2 exon 3 boundary of a normal weight individual (B) and an obese patient with a hypermethylated POMC variant (C). (PDF) [file pgen.1002543.s001.pdf]
