# Supplementary figures and images for: An Alu Element–Associated Hypermethylation Variant of the POMC Gene Is Associated with Childhood Obesity
Source: PLoS Genet. 2012 Mar 15;8(3):e1002543. doi: 10.1371/journal.pgen.1002543 (PMC3305357; doi:10.1371/journal.pgen.1002543)

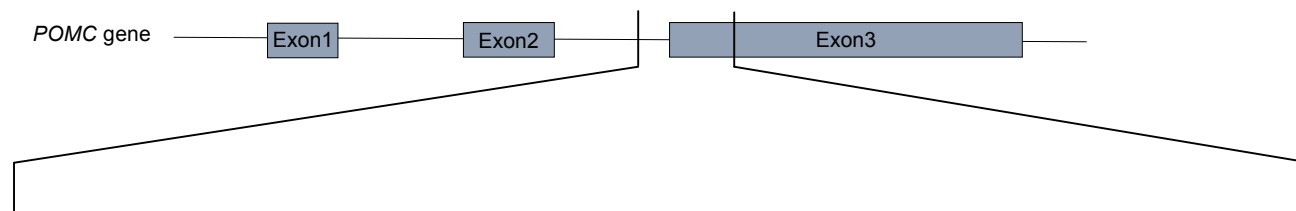

Normal weight individual No.1

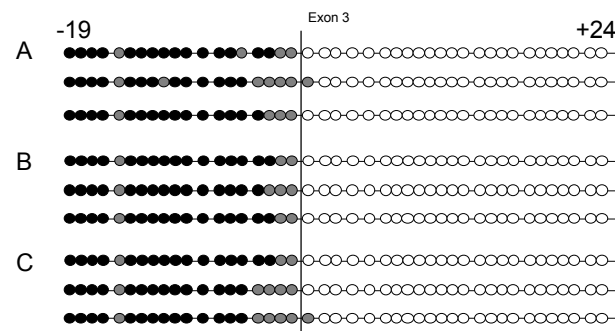

Obese patient No.1

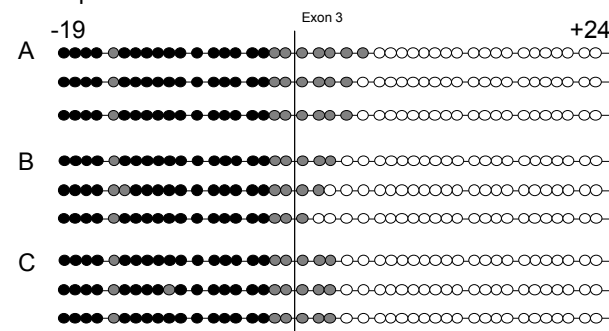

Normal weight individual No.2

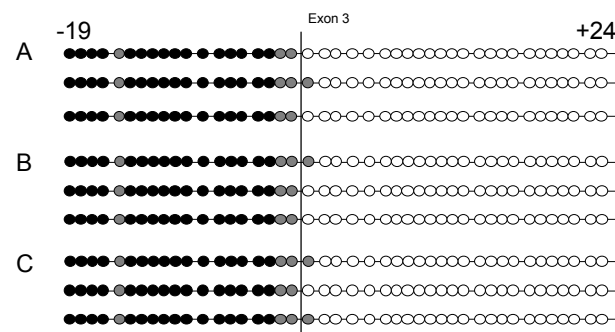

Obese patient No.2

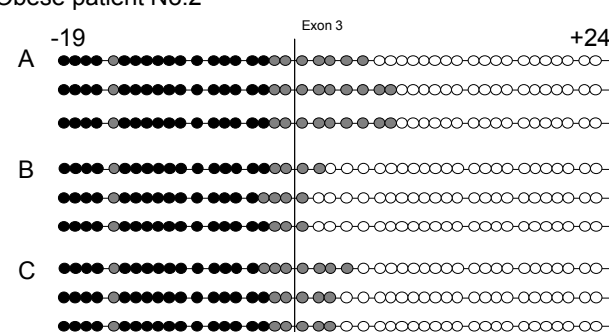

Supplement: Figure S2 — Analysis of the intra-individual variability of the POMC DNA methylation at the intron2-exon3 intersection. DNA methylation was analysed from two normal weight individuals and two obese individuals with the hypermethylated variant. The DNA was extracted from three different blood samples of each individual (A, B, C) and three independent PCR amplification reactions were performed with each DNA sample. (PDF) [file pgen.1002543.s002.pdf]

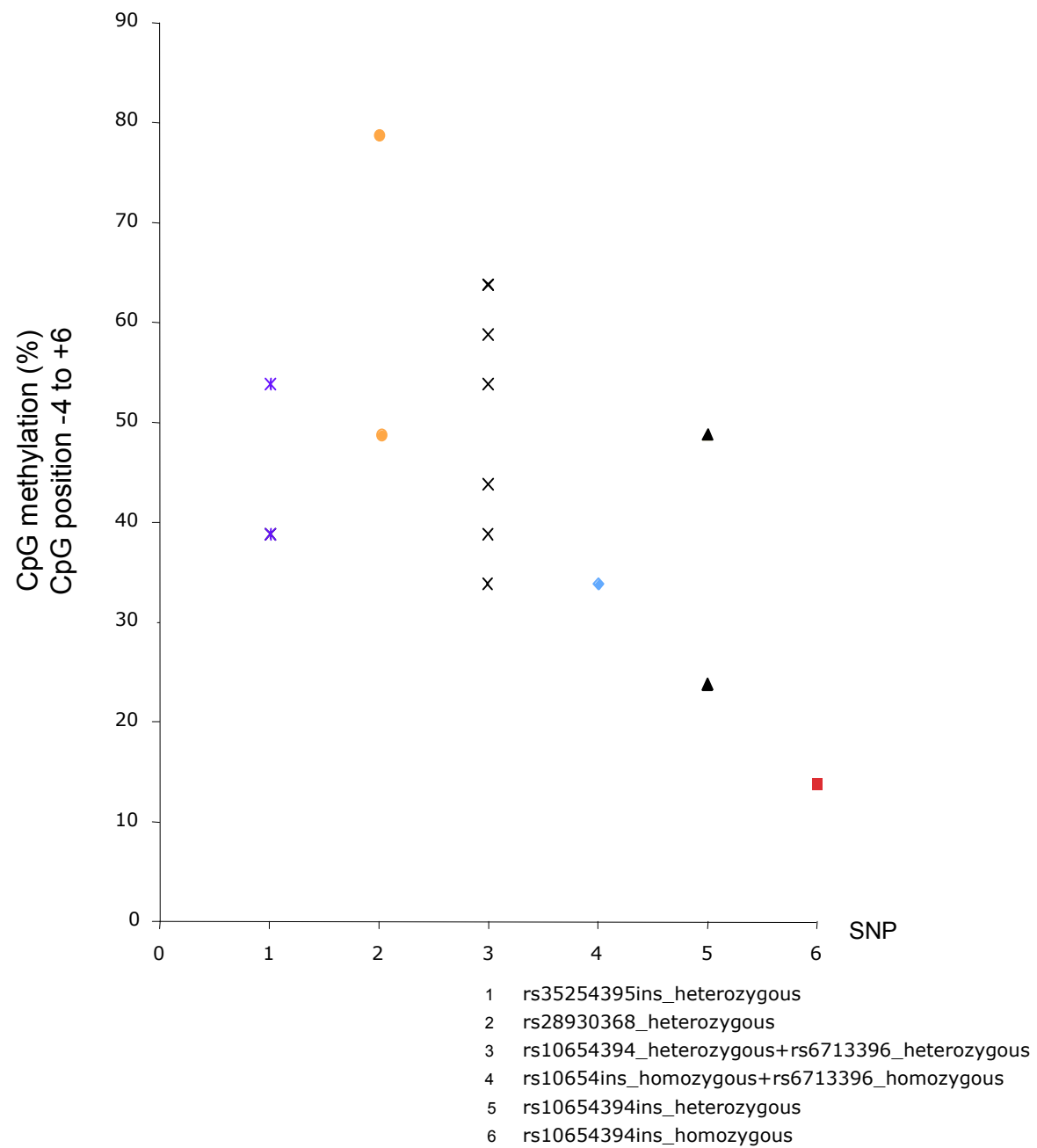

Supplement: Figure S3 — Diagram of the observed SNPs (No1-6) within the POMC gene in correlation to the observed DNA methylation intensity at CpG position −4 to +6 of the individual respectively. (PDF) [file pgen.1002543.s003.pdf]

**A**

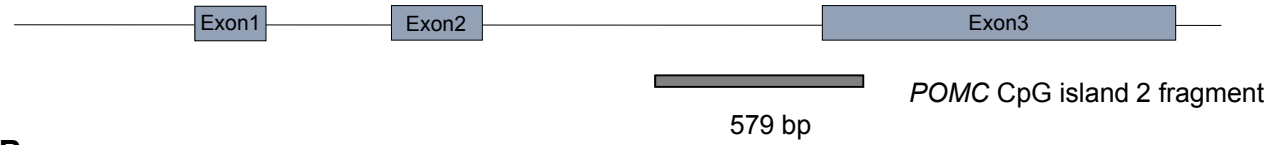

**B**

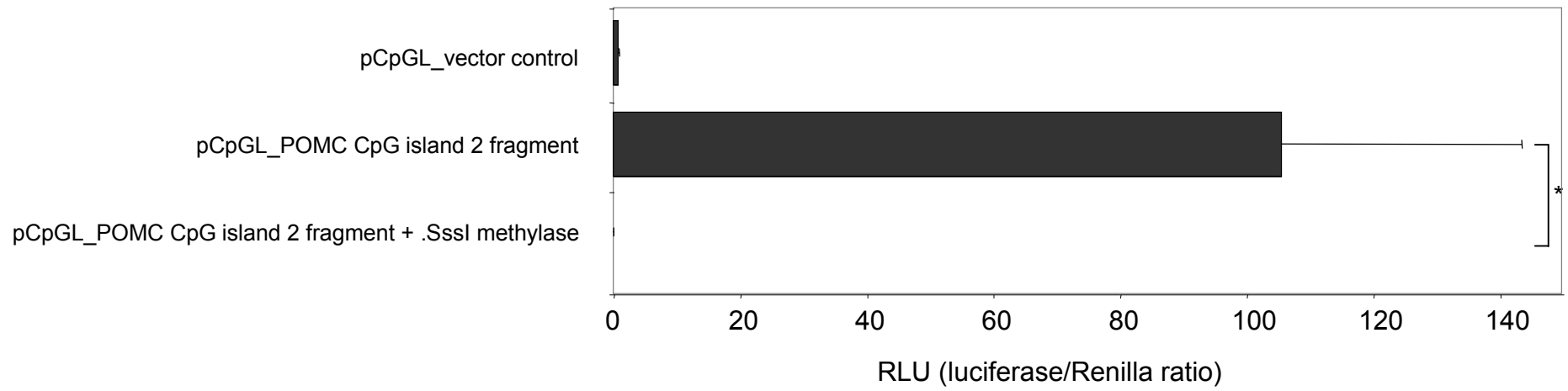

Supplement: Figure S4 — Luciferase reporter-gene assay with a POMC CpG island 2 fragment. A schematic display of the fragment localization B Luciferase assay with empty pCpGL vector, pCpGL_CpG island 2 vector treated with Sss1 or treated with Sss1 without SAM addition as a control. The luciferase activity is annotated according the luciferase/renilla ratio of 3 independent experiments. (PDF) [file pgen.1002543.s004.pdf]

genom. IP Prä-IP neg  
Marker DNA DNA DNA control

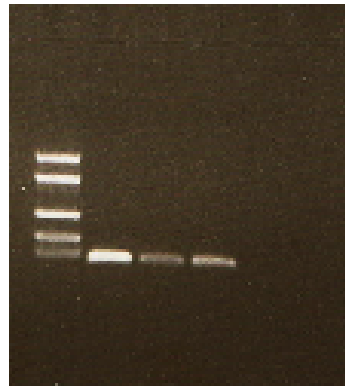

Supplement: Figure S5 — P300 ChIP analysis of the insulin promoter with samples of β-TC3 beta-cells. We used this published P300 binding site [34] as positive control for the established ChIP assay. (PDF) [file pgen.1002543.s005.pdf]
