# Supplementary material for: An Alu Element–Associated Hypermethylation Variant of the POMC Gene Is Associated with Childhood Obesity
Source: PLoS Genet. 2012 Mar 15;8(3):e1002543. doi: 10.1371/journal.pgen.1002543 (PMC3305357; doi:10.1371/journal.pgen.1002543)
Supplement: Text S1 — A summary of the different bisulphite sequencing protocols used in this study. Moreover the primer pair sequences and the different protocols for ChIP and qPCR experiments are described in detail. (DOC) [file pgen.1002543.s006.doc]

**Text S1**

**An Alu element associated hypermethylated variant of the *POMC* gene is associated with childhood obesity**

Peter Kuehnen, Mona Mischke, Susanna Wiegand, Christine Sers, Bernhard Horsthemke, Susanne Lau , Thomas Keil, Young-Ae Lee, Annette Grueters, Heiko Krude

DNA methylation analysis with agarose beads:

Bisulphite modification was performed according to standard protocols . DNA (500 ng) was digested with 20 IE Eco RI for 2 hours at 37 °C and denatured 5 min at 95 °C. Samples were incubated for alkaline denaturation with 4 µl NaOH 2 M (Merck) for 15 min at 50 °C. Bisulphite solution was prepared with 1.9 g sodium bisulphite (Merck), 2.5 ml H_2_O, 750 µl NaOH 2M and 5 mg hydroquinone (Sigma). The bisulphite solution was covered with mineral oil (Sigma). The denaturized DNA was mixed with 50 µl melted LMP agarose (2 %). 10 µl of this mix was transferred into the oil supernatant. Thereby DNA agarose-beads were created and transferred into the bisulphite solution beneath the oil phase. Samples were incubated at 50 °C for 8 hours. After this incubation DNA agarose beads were washed with 1 x TE (Sigma) 3 x 10 min, NaOH (0.2 M) 2 x 15 min and finally 1 x TE 3 x 10 min. Samples were amplified by PCR. This product was analyzed by direct sequencing or sequencing after cloning with the 2.1 Topo cloning kit (Invitrogen) of 10-20 clones.

For the analysis of human brain samples beta-MSH positive cells 50-100 per sample were extracted by laser microdissection (ZEISS) and treated with bisulphite solution according published protocols . The methylation levels of each CpG position was quantified by dividing the number of methylated CpG positions by the total number of cloned sequences.

DNA methylation analysis without agarose beads:

1 μg DNA extracted from peripheral blood cells were treated with sodium bisulphite according to modificated standard protocols . DNA was denatured with NaOH (3M) and incubated for 15 min at 37°C. DNA was heated to 95°C for 2 min as a second denaturating step. Samples were added to fresh prepared bisulphite solution with 1.9 g sodium bisulphite (Merck), 2.5 ml H_2_O, 750 µl NaOH (2M) and 5 mg hydroquinone (Sigma). After incubation for 12 h at 50°C samples were washed with the Wizard DNA clean up system (Promega) and DNA was extracted. After PCR direct sequencing reaction was performed with automatic sequencing (ABI 3100, Applied Biosystems). The CpG methylation intensity was quantified by modification of described protocols . In the bisulphite-converted sequence a single cytosine was considered to be 100 % and single thymine to be not methylated. Furthermore overlapping cytosines and thymines at a CpG position was considered to be partial methylated and calculated as 50 %. Method accuracy was examined by analysis of 6 samples each with both bisulphite-methods (agarose-beads based and without agose-beads), in which the DNA methylation pattern was reproducible (data not shown).

Primer sequences:

Oligos (TibMolBiol):

| Name | primer sequence |
| --- | --- |
| POMC F1 forward out | 5´-TTT TAA AGT GGA ATA GAG AGA ATA TGA-3´ |
| POMC F1 forward-in | 5´-GTG GAA TAG AGA GAA TAT GAT TTT TT-3´ |
| POMC F1 reverse-out | 5´-CTC CTC TAT CCT TAT ATA CTT ACC-3´ |
| POMC F1 reverse-in | 5´-ACA ACA CAA AAA ACA ACA CCC C-3´ |
| POMC F2 forward | 5´- GTA AGT ATA TAA GGA TAG AGG AG-3´ |
| POMC F2 reverse-out | 5´-CTC TCC AAC ATA AAC AAC AAA AAC-3´ |
| POMC F2 reverse-in | 5´- AAT TAT CCC AAA ACC TCC TAA CAA-3´ |
| POMC F3 forward-out | 5´-TTA GAT AAA TTA TGG AAT GGG A-3´ |
| POMC F3 forward-in | 5´-GTG GTA AGA TTT TAG ATG TTT-3´ |
| POMC F3 reverse-out | 5´-AAA CTC CAA AAA AAA AAC CTC-3´ |
| POMC F3 reverse-in | 5´-AAA ATA CTC CAT AAA ATA AAA AC-3´ |
| Mouse POMC F1 forward-out | 5´-GGT ATA GAA GGA TAT TTG TTT TGA AAT A-3´ |
| Mouse POMC F1 forward-in | 5´-TTT ATT TTA AAA GGT AGT TTG TTT TGG G-3´ |
| Mouse POMC F1 reverse-out | 5´-TCC ACT TAA AAC TAA ACA AAA ACT TAA C-3´ |
| Mouse POMC F1 reverse-in | 5´-CAA ACC TAA TTC TAA AAT CTT ACA AAT C-3´ |
| Mouse POMC F2 forward-out | 5´-GAT TTG TAA GAT TTT AGA ATT AGG TTT G-3´ |
| Mouse POMC F2 forward-in | 5´-GTT AAG TTT TTG TTT AGT TTT AAG TGG A-3´ |
| Mouse POMC F2 reverse-out | 5´-CCC ATC TCA AAA ATT TAA AAA AAA ATC AA-3´ |
| Mouse POMC F2 reverse-in | 5´-CCA ATC TAC TAA AAA TCC CAA AAT CC-3´ |
| Mouse POMC F3 forward-out | 5´-GTT TAT GTT TGT TTT GGA TTT AAA TAG-3´ |
| Mouse POMC F3 forward-in | 5´-GTA AGA TTT TGT TAG TAA GAG TTA AG-3´ |
| Mouse POMC F3 reverse | 5´-CAA CAC TAC TAT TCC TAA AAC-3´ |
| Mouse POMC F4 forward-out | 5´-GAG ATG AAT AGT TTT TGA TTG AAA AT-3´ |
| Mouse POMC F4 forward-in | 5´-GTT TTA GGA ATA GTA GTA GAG TTG-3´ |
| Mouse POMC F4 reverse-out | 5´-CCT AAC ACA AAT AAC TCT AAA AAA C-3´ |
| Mouse POMC F4 reverse-in | 5´-CCC ATA CAA AAA AAA AAC CTT AAA AT-3´ |
| Alu_F_forward_out | 5´- GGG TTT TAG TAG TTA GAT TTT TAT- 3´ |
| Alu_F_reverse-out | 5´- ACC AAA ATC TTA CTA TTA TAT ACC- 3´ |
| Alu_F_forward-in | 5´- GTT AGA TTT TTA TTA TTT GGA GAG- 3´ |
| Alu_F-reverse-in | 5´- TAC CTA ACA TAA AAA AAT AAC TCC- 3´ |

Postmortem human brain tissue and immunohistochemistry

Human brain samples (cyrofixed) were cut into 10 µm sections and stained with the beta-MSH antibody (G. Brabant, Birmingham, GB). Immunohistochemical analysis of Beta-MSH of human arcuate nucleus was carried out as previously described . Sections were mounted on slides and fixed overnight at 37 °C. Samples were incubated with 3 % hydrogen peroxide in methanol for 15 minutes at room temperature. During washing steps the samples were incubated with a 1:20 dilution of normal goat serum in PBS for 20 min and then incubated with the polyclonal mouse anti human beta-MSH antibody 1:100 in 10 % fetal bovine serum in PBS. Section were washed and treated with a goat anti-mouse IgG (DAKO; 1:100). For visualization of beta-MSH positive cells, sections were treated with the Vectastain ABC Elite Kit (Vector Laboratories) and diaminobenzidine (Sigma). Beta-MSH positives cells of the arcuate nucleus (50-100 per sample) were extracted by laser microdissection (ZEISS).

Chromatin immune-precipitation (ChIP)

Peripheral blood cells were extracted by Bicoll (BioRad) from 40 ml EDTA blood and harvest in culture medium (DMEM, 5%P/S, 5% L-Glutamine, 10 % FBS). Cells were treated with 1% formaldehyde according to standard protocols . Chromatin was extracted after glycine treatment and samples were sonicated (12x15 sec, power 50%). Thereafter samples were preincubated with magnetic beads (Invitrogen) for 1 h (+4°C). Finally probes were transferred to P300 antibody (N-15, Santa Cruz) labelled magnetic beads and incubated overnight on a tilting table. After washing steps samples were incubated for 16 hours at 65°C and final PCR was performed with two different primer pairs after ethanol washing steps. Furthermore samples were also incubated with magnetic beads and anti-FLAG antibody (SIGMA) as well as non-antibody labelled magnetic beads as negative control experiments. Quantitative real time PCR reactions were performed using iCycler IQ (BioRad) and added SYBR green mix (ABgene) with the second primer pair (POMC_2_ChIP) in triplets. The relative proportion of ChIP PCR products were evaluated as percent recovery in relation to the appropriate pre-IP sample.

| Name | primer sequence |
| --- | --- |
| POMC_1_ChIP forward | 5´- GGC CTA GGC GCA GTG AC -3´ |
| POMC_1_ChIP reverse | 5´- GAA GTG GCC CAT GAC GTA CT-3´ |
| POMC_2_ChIP forward | 5´- GGT GCG GGG CAC GGG C -3´ |
| POMC_2_ChIP reverse | 5´- TTC CGG GGG TTC TCG GTC -3´ |
| INS_prox_for | 5´- TCA GCC AAA GAT GAA GAA GGT CTC -3´ |
| INS_prox_rew | 5´- TCC AAA CAC TTG CCT GGT GC -3´ |

Luciferase reportergene-assay

The CpG free pCpGL vector was obtained from AG Rehli (Regensburg, Germany) .

Primer sequence for intron2_exon3 fragment (including restriction recognition site):

| POMC_CpGL_for | 5´- CGA TGG ATC CGC AGT TGC CAG GCA GAA GCA -3 |
| --- | --- |
| POMC_CpGL_rew | 5´- CGA TCC ATG GAG TAG GAG CGC TTG CCC TCG -3 |

cDNA synthesis and POMC real time PCR

qPCR was performed according standard protocols using the iCycler system (Biorad). Data was analysed after the ∆∆CT method .

| Name | primer sequence |
| --- | --- |
| ß-actin forward | 5´- CTC TTC CAG CCT TCC TTC CT -3´ |
| ß-actin reverse | 5´- AGC ACT GTG TTG GCG TAC AG -3´ |
| POMC_RT forward | 5´- TTC AAG AGG GAG CTG ACT GG-3´ |
| POMC_RT reverse | 5´- TTC TCG GAG GTC ATG AAA CC-3´ |
| POMC_RT_exon2_3_for | 5´- CAG GAC CTC ACC ACG GAA AGC AAC C -3´ |
| POMC_RT_exon2_3_rew | 5´- CAC CTT CAC TGG GCG CCG CTT CTT G -3´ |

**References**:

1. Hajkova P, el-Maarri O, Engemann S, Oswald J, Olek A, et al. (2002) DNA-methylation analysis by the bisulfite-assisted genomic sequencing method. Methods Mol Biol 200: 143-154.

2. Kerjean A, Vieillefond A, Thiounn N, Sibony M, Jeanpierre M, et al. (2001) Bisulfite genomic sequencing of microdissected cells. Nucleic Acids Res 29: E106-106.

3. Clark SJ, Statham A, Stirzaker C, Molloy PL, Frommer M (2006) DNA methylation: bisulphite modification and analysis. Nat Protoc 1: 2353-2364.

4. Eckhardt F, Lewin J, Cortese R, Rakyan VK, Attwood J, et al. (2006) DNA methylation profiling of human chromosomes 6, 20 and 22. Nat Genet 38: 1378-1385.

5. Biebermann H, Castaneda TR, van Landeghem F, von Deimling A, Escher F, et al. (2006) A role for beta-melanocyte-stimulating hormone in human body-weight regulation. Cell Metab 3: 141-146.

6. Kuo MH, Allis CD (1999) In vivo cross-linking and immunoprecipitation for studying dynamic Protein:DNA associations in a chromatin environment. Methods 19: 425-433.

7. Klug M, Rehli M (2006) Functional analysis of promoter CpG methylation using a CpG-free luciferase reporter vector. Epigenetics 1: 127-130.

8. Livak KJ, Schmittgen TD (2001) Analysis of relative gene expression data using real-time quantitative PCR and the 2(-Delta Delta C(T)) Method. Methods 25: 402-408.
